# Sleep Quality and Associated Lifestyle Factors Among Medical Students Before and After the COVID-19 Era—A Comparative Study from Romania

**DOI:** 10.3390/medicina62050880

**Published:** 2026-05-04

**Authors:** Bogdana Adriana Năsui, Lorena Gorgan, Codruța Alina Popescu, Nina Ciuciuc, Alexandra-Ioana Roșioară, Dana Manuela Sîrbu, Monica Popa, Daniela Curșeu, Ileana Monica Borda, Rodica Ana Ungur

**Affiliations:** 1 Department of Community Medicine, Iuliu Hațieganu University of Medicine and Pharmacy, 400349 Cluj-Napoca, Romania; adriana.nasui@umfcluj.ro (B.A.N.); gorganlorena@gmail.com (L.G.); nina.ciuciuc@umfcluj.ro (N.C.); alexandra.rosioara@umfcluj.ro (A.-I.R.); dsirbu@umfcluj.ro (D.M.S.); monica.popa@umfcluj.ro (M.P.); dcurseu@umfcluj.ro (D.C.); 2 Research Center in Preventive Medicine, Health Promotion and Sustainable Development, Iuliu Hațieganu University of Medicine and Pharmacy, 400349 Cluj-Napoca, Romania; 3 Department of Human Sciences, Iuliu Hațieganu University of Medicine and Pharmacy, 400012 Cluj-Napoca, Romania; 4 Department of Medical Specialties, Faculty of Medicine, Iuliu Hațieganu University of Medicine and Pharmacy, 8 Victor Babeș Street, 400012 Cluj-Napoca, Romania; monica.borda@umfcluj.ro (I.M.B.); rodica.ungur@umfcluj.ro (R.A.U.)

**Keywords:** sleep quality, nutritional status, alcohol consumption, illicit drugs, physical activity, risky behaviors, wellbeing, lifestyle choices, diet, students

## Abstract

*Background and Objectives:* Sleep is a vital psychological function for health and well-being in all age groups, from children to adolescents, to adults and the elderly, and impacts quality of life. This study evaluated temporal changes in sleep quality and lifestyle behaviors among medical students in North-Western Romania (Transylvania) between the COVID-19 pandemic and the post-pandemic period. *Materials and Methods:* A cross-sectional design was employed involving 709 medical students assessed during the first pandemic wave (2020) and the 2023–2024 academic year. Online questionnaires collected data on demographics, body mass index (BMI), substance use, and physical activity. Sleep quality was measured using the validated Athens Insomnia Scale (AIS), and multiple linear regression was performed to identify predictors of sleep outcomes. *Results:* Post-pandemic data revealed a significant decline in sleep quality, with female gender and lower academic performance identified as significant predictors of insomnia symptoms (R^2^ of 0.258, *p* < 0.05). While physical activity levels improved significantly in 2024 compared to the confinement period, this was accompanied by increased fast-food consumption and a rise in overweight and obesity rates. Conversely, illicit drug use decreased, and alcohol consumption patterns shifted, characterized by reduced weekly frequency among females but persistent binge drinking episodes. *Conclusions:* The transition to post-pandemic education yielded mixed health outcomes; while physical activity rebounded, sleep quality and nutritional status deteriorated. These findings highlight the necessity for university-based interventions focusing on sleep hygiene, nutrition, and stress management to support the well-being of medical students.

## 1. Introduction

Sleep is a vital psychological function for health and wellbeing in all age groups from children and adolescents to adults and the elderly, and impacts the quality of life, affecting the cardiovascular, cerebrovascular and metabolic systems. Insufficient sleep in adults is defined as less than 7 h of sleep in 24 h. The Center for Disease Control and Prevention estimates that, as a nation, 35% of adults do not get enough sleep [1,2]. Sleep disorders have been found to be associated with weight disorders, obesity and metabolic syndrome [3]. Moreover, quality of sleep is associated with mental health disorders, and these conditions are public health challenges because both have an important impact on individual and global wellness [4].

The university years are marked by changes in living arrangements, academic responsibilities, social obligations and newfound independence. Following these transitional challenges, increased psychological distress and diminished well-being have become widespread, representing significant public concern [5].

Sleep quality is crucial for students’ success and impacts academic performance, cognitive function and overall well-being. A good sleep quality contributes to memory consolidation, learning, problem-solving and ultimately leads to good academic outcomes and reduced stress [6,7]. Medical students carry a large academic load, which could potentially contribute to poor sleep quality. Poor sleep quality is also common among non-medical students and in the general population in modern society [8].

The pandemic negatively impacted people’s sleep across the world. During the COVID-19 pandemic young adults reported experiencing anxiety, depression and a high level of stress that could directly influence sleep quality. On 12 January the World Health Organization confirmed a coronavirus outbreak [9]. In Romania, on 12 March, the president declared a state of emergency. During this period, the schools were closed; however, the activities were carried out online. Also, the emergency included the closure of restaurants, clubs, and hotels, the gradual closure of borders, or limiting or prohibiting the movement of vehicles or people in/to certain areas [10,11].

Data from the literature regarding the quality of sleep during the pandemic is controversial. Factors that influenced sleep quality were a high level of COVID-19 stress, caffeine consumption, urban residence, nuclear family, and current smokers [12]. Social isolation during the lockdown led to moderate symptoms of anxiety, and, consequently, poor quality of sleep, especially among female students [13,14]. On the other hand, COVID-19 training, high level of resilience and COVID-19 history were negatively associated with sleep quality [15].

There are a few studies that investigated the quality of sleep among the Romanian general population [16,17,18] or adolescents [19]. None of them made a comparison between the pandemic and the post-pandemic period. The study aimed to assess the quality of sleep and associated lifestyle factors among medical students from the North-western part of Romania during two different periods, the pandemic and post-pandemic period.

Medical students represent a particularly vulnerable subgroup within the university population. Compared to students from other academic fields, they are exposed to sustained academic pressure, competitive environments, long study hours, and demanding curricula [20]. These factors have been consistently associated with higher rates of stress, anxiety, depression, and burnout among medical students [21,22], which may directly and indirectly impair sleep quality [23,24]. Moreover, heavy academic workload, examination stress, and clinical training demand further contribute to sleep disturbances and reduced sleep duration [25]. Therefore, investigating sleep patterns specifically in medical students is essential for understanding risk mechanisms in a high-pressure academic context.

While most studies have focused on the immediate psychological and behavioral consequences of COVID-19 confinement, there is limited evidence regarding the medium-term evolution of sleep quality and lifestyle behaviors in the post-pandemic period. This gap is particularly evident in underrepresented Eastern European populations. Therefore, this study aims to examine changes in sleep quality and associated lifestyle factors among medical students across the pandemic and post-pandemic periods.

## 2. Materials and Methods

To achieve the objectives, a cross-sectional study was performed. The sample consisted of 709 medical students from medical schools in the North-Western part of the country, Transylvania. The survey was initially administered during the first wave of the COVID-19 pandemic (April–May 2020) and subsequently re-administered during the 2023–2024 academic year (April–May 2024). Using this approach, a convenience sampling method was applied. Informed consent was obtained from the students by having them fill out the questionnaires online. We distributed the questionnaire through Google Forms (Google LLC, Mountain View, CA, USA). This study was approved by the Ethics Committee of the Cluj Napoca University of Medicine and Pharmacy (Approval No. 45/2 May 2019, Approval No. 179/20 September 2024).

The questionnaire comprised questions that investigated:

- Demographic data: age, place of living, marital status, sex, year of study, academic performance, weight (kg), height (m)

Living arrangements and marital status were recorded using predefined categorical response options.

Body mass index (BMI, kg/m^2^) was calculated based on self-reported height and weight and categorized as underweight (BMI < 18.5 kg/m^2^), normal weight (BMI: 18.5–24.9 kg/m^2^), overweight (BMI: 25–29.9 kg/m^2^), and obese (BMI > 30 kg/m^2^), according to WHO categories.

b. Quality of sleep

To assess sleep quality among Romanian medical students, we employed the Athens Questionnaire [26], which was translated into Romanian and validated as part of the present study. In addition to its high internal consistency, the Athens Insomnia Scale (AIS) has demonstrated good construct and criterion validity, having been originally validated against clinical diagnoses of insomnia based on ICD-10 criteria [26]. The instrument has shown satisfactory sensitivity and specificity for detecting insomnia in both clinical and general populations [26,27]. Subsequent studies conducted in different cultural contexts have confirmed its cross-cultural validity and robustness as a screening tool for insomnia [27,28,29], supporting its use in diverse populations. The Romanian version of the scale was analyzed using factor analysis, with principal component analysis applied as the extraction method and an eigenvalue threshold of 1.0. Item-total correlations were assessed using Pearson’s correlation coefficients. The mean item-total correlation values were high, and the factor analysis indicated that the scale loaded onto a single component. AIS consists of 8 items: nocturnal symptoms (sleep induction, awakening during the night, final awakening earlier than desired), total sleep duration, overall quality of sleep, and daytime disfunctions (sense of well-being during the day, functioning (physical and mental) during the day, and sleepiness during the day). Each item is rated on a 4-point Likert scale ranging from 0 (no problem at all) to 3 (very serious problem). The total score ranges from 0 to 24, with higher scores indicating greater severity of insomnia symptoms. A total score of ≥6 was used as the cut-off value to indicate the presence of insomnia, consistent with previous validation studies. Participants were stratified into two groups based on the Athens Insomnia Scale: students without insomnia (score < 6) and students with insomnia (score ≥ 6).

Furthermore, the translation and back-translation procedure applied in the present study contributes to ensuring the content validity and linguistic equivalence of the Romanian version, in accordance with established methodological recommendations for cross-cultural adaptation of psychometric instruments [30]. To ensure linguistic equivalence, a forward–backward translation procedure was applied. Two independent translators translated the questionnaire into Romanian, followed by back-translation into English. The Romanian version was pretested on 30 respondents to evaluate clarity and feasibility. Internal consistency was evaluated using Cronbach’s alpha, which demonstrated excellent internal consistency (α = 0.88), comparable to the original English version.

In addition to total AIS scores, item-level responses were analyzed separately by gender and survey year. Sleep duration was assessed as self-reported average number of hours slept during academic/exam sessions and outside academic/exam sessions.

The questionnaire also examined associated healthy or unhealthy behaviors among students.

c. The questionnaire investigated alcohol consumption among university students. Alcohol consumption was estimated as frequency and as the number of drinks consumed in a row by the students. In addition, excessive drinking among students was assessed as more than 5 drinks in a row per month. The frequency of binge drinking episodes per month was additionally recorded and categorized as none, once, 2 times, 3–5 times, 6–9 times, and more than 10 times per month.
d. Additional unhealthy behaviors assessed in the study included illicit drug use, smoking, and fast-food consumption. Illicit drug use and smoking were measured using self-reported dichotomous (yes/no) items.

Fast food intake was evaluated as frequency: never, sometimes, 1–3 times/week, 4–6 times per week, every day.

e. Physical activity was assessed using self-reported frequency of walking, moderate-intensity physical activity, and vigorous-intensity physical activity. Participants were asked to report how often they engaged in: (1) walking for at least 30 min per day, (2) moderate physical activity lasting at least 10 min, and (3) vigorous physical activity lasting at least 10 min.

For each activity domain, response options were categorized into four frequency groups: never, 1–3 times per week, 4–6 times per week, and every day. Analyses were stratified by sex and survey year (2020 and 2024). Differences in physical activity patterns between years were examined separately for males and females.

We calculated the representative sample based on the student population (approximately 9752) with a margin of error of % 5 and a 95% confidence level [31]. The estimated sample size was 370 respondents. Sampling was performed at the level of academic series (teaching groups). From the total population of students, teaching groups were randomly selected within each year of study. All students within the selected teaching groups were invited to participate via the institutional Microsoft Teams platform of the teaching group where the questionnaire in Google Forms was distributed.

**Figure 1:**
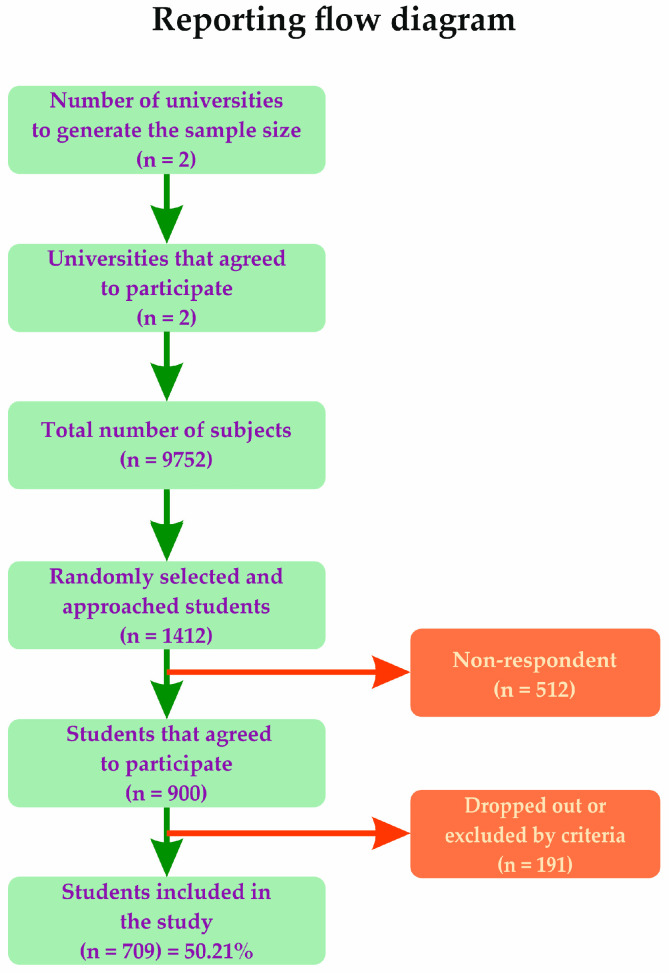
Flow diagram of sample size selecting process.

The survey was distributed to 1412 students, yielding an overall response rate of approximately 50%. Following the application of predefined exclusion criteria, 709 students were included in the final analysis, as illustrated in the flow diagram (Figure 1). Inclusion criteria: medical students of all ages and genders who agreed to participate in the study via online informed consent; exclusion criteria: preexisting sleep disorders, incomplete responses to the questionnaires provided.

### Statistical Analysis

Descriptive statistics were calculated to summarize participants’ sociodemographic and lifestyle characteristics. Categorical variables (e.g., gender, academic performance, living arrangements, smoking, alcohol and fast-food consumption, and physical activity) were presented as frequencies and percentages, while continuous variables (e.g., age, sleep duration, BMI, and Athens Insomnia Scale scores) were expressed as means ± standard deviations (SD). All analyses were stratified by sex and survey year (2020 and 2024), where applicable.

The Chi-square (χ^2^) test was applied to assess associations between categorical variables, such as gender, academic year, lifestyle behaviors, and survey year (2020 vs. 2024). The analysis of variance (ANOVA) was used to compare mean differences for continuous variables, including sleep duration, Athens’s insomnia scores, and BMI across groups. Two-way analysis of variance (ANOVA) was used to examine the main and interaction effects of gender and survey year (2020 vs. 2024) on continuous sleep-related outcomes

Assumptions of normality and homogeneity of variances were verified using the Shapiro–Wilk and Levene’s tests, respectively. A multiple linear regression analysis was conducted to identify predictors of sleep quality (Athens Insomnia Scale score), with academic performance, alcohol consumption, illicit drug use, and gender entered as independent variables. Statistical significance was set at *p* < 0.05. Analyses were performed using IBM SPSS Statistics version 26.0 (IBM Corp., Armonk, NY, USA).

## 3. Results

A total of 709 participants was included in the 2020 and 2024 surveys. The 2020 survey included N = 384 participants, and the 2024 survey included N = 325 participants. The proportion of male students was 23.7% (N = 91) in 2020 to 20.3% (N = 83) in 2024. The mean age of the participants was 21.50 ± 2.06 years (in 2020, the mean age was 21.45 ± 2.28, and in 2024, the mean age was 21.57 ± 1.78 years). Most participants were female across both survey years.

Although academic performance showed a slight improvement in 2024, with the proportion of students reporting “excellent” results increasing from 7.3% to 11.7%, the majority of participants in both surveys described their academic results as good. Most participants lived in student dormitories in 2024 (42.8%), compared with 15.1% in 2020. The percentage of students living with their parents decreased substantially over time (43.8% in 2020 vs. 11.7% in 2024).

Most respondents were single, with a significant increase in the proportion of single students between 2020 (51.3%) and 2024 (75.4%) (*p* < 0.001). Table 1 presents general characteristics of the sample size. Investigating the body mass index distribution among students, the results of the study revealed that the proportion of normal weight students decreased (74% vs. 68.3%) while the distribution of students with overweight (10.9% vs. 19.1%) (*p* < 0.05) increased during the post-pandemic period (Table 1).

### 3.1. Quality of Sleep

Mean sleep duration during academic sessions changed between 2020 and 2024, from 7.27 ± 0.93 h (2020) to 7.29 ± 0.97 h (2024) in males and from 6.77 ± 1.55 h (2020) to 6.50 ± 1.35 h (2024) in females (*p* = 0.08). Additionally, sleep duration outside the academic session also declined significantly, from 7.45 ±1.35 h (2020) to 7.12 ± 1.33 h (2024) in males and from 6.77 ± 1.23 h (2020) to 7.61 ± 4.47 h (2024) in females. (*p* = 0.009) (Table 2).

A two-way ANOVA was conducted to examine the effects of gender and year on sleep quality scores. The Athens Insomnia Scale scores increased in both genders over time, from 4.05 ± 3.09 to 4.45 ± 3.16 in males and from 4.50 ± 3.09 to 5.49 ± 3.18 in females.

There was a significant main effect of gender, F = 7.347, *p* = 0.007, indicating that overall sleep quality differed between boys and girls.

There was also a significant main effect of year, F = 6.505, *p* = 0.011, with overall sleep quality scores differing between 2020 and 2024 and indicating a deterioration in sleep quality.

The interaction between gender and year was not statistically significant, F = 1.165, *p* = 0.281, suggesting that the difference in sleep quality between boys and girls did not change significantly from 2020 to 2024.

A two-way ANOVA was conducted to examine the effects of gender and year on hours of sleep during the exam session.

The analysis showed no significant main effect of gender, F = 1.450, *p* = 0.229, indicating that males and females did not differ significantly in average sleep duration during exams.

There was a significant main effect of year, F = 38.969, *p* < 0.001, suggesting that average sleep hours during the exam session differed between 2020 and 2024.

The interaction between gender and year was not statistically significant, F = 1.964, *p* = 0.162, indicating that the change in sleep duration across years was similar for both genders.

A two-way ANOVA was conducted to examine the effects of gender and year on hours of sleep outside the exam session. The analysis revealed no significant main effect of gender, F = 0.159, *p* = 0.690, indicating that average hours of sleep did not differ significantly between males and females.

Similarly, there was no significant main effect of year, F = 1.066, *p* = 0.302, suggesting no overall difference in hours of sleep between 2020 and 2024.

However, there was a significant interaction between gender and year, F = 5.622, *p* = 0.018, indicating that the relationship between gender and hours of sleep varied across the two years.

Although the main effect of year was not statistically significant, the significant gender × year interaction indicates that changes in sleep duration outside the exam session differed between males and females over time.

The mean Athens score increased during the post-pandemic period, especially among females (*p* = 0.01) (Table 2).

Moreover, a large percentage of students, especially females, are not waking rested in the morning, suggesting low-quality sleep (Table 3).

We investigated the prevalence of insomnia among medical students using the Athens Insomnia Scale (AIS). Among males, the proportion of participants with an AIS score < 6 decreased from 80.2% (N = 73) in 2020 to 73.5% (N = 81) in 2024, while those with an AIS score ≥ 6 increased from 19.8% (N = 18) to 26.5% (N = 22). Similarly, among females, the prevalence of AIS scores ≥ 6 increased from 22.2% (N = 65) to 30.6% (N = 74). However, differences between 2020 and 2024 were not statistically significant (*p* > 0.05) (Table 4). The detailed scores for Athens Insomnia Scale (AIS) are in supplementary Table S1.

The present study analyzed in detail each parameter of the Athens Insomnia Scale. The quality of sleep was slightly unsatisfactory for the majority of female students after the pandemic (46.7% vs. 37.2%). During the post-pandemic period, 10.7% of female students reported considerable problems with awakening during the night (*p* = 0.002). Overall sleep quality declined among females after the pandemic period. Sense of well-being during the day also decreased in 2024, while daytime sleepiness increased during the period of in-person learning (*p* = 0.004). 

### 3.2. Alcohol Consumption

Regarding alcohol consumption, the study revealed that the proportion of respondents who were abstinent increased from 2020 to 2024, both in males and females. In males, the students who reported the alcohol intake 1–4 times per week decreased from 74.7% (N = 68) to 66.3% (N = 55). Overall, no statistically significant change in alcohol consumption patterns was observed among males between 2020 and 2024 (*p* = 0.31). Similarly, the female students reported a decrease in alcohol consumption with a frequency of 1–4 times per week (64.5% vs. 50.4%). The overall change in alcohol consumption distribution among females was statistically significant (*p* = 0.002) (Table 5).

The study also investigated the amount of alcohol consumption among students as the number of drinks consumed in a row. The results of the study show that the majority of students, both male and female, drank 1–3 drinks per row. On the other hand, the proportion of males who reported drinking 4–7 drinks in a row decreased from 35.2 to 21.7%. Similar trends were observed in female students; the consumption of 4–7 drinks in a row decreased after the pandemic period (18.1% vs. 11.2%) (*p* < 0.05) (Table 5).

Binge drinking patterns remained relatively stable among males, while increased engagement was observed among females. High-frequency binge drinking episodes (>10 times/month) remained rare.

The percentage of females reporting no binge drinking episodes decreased from 75.1% (n = 220) in 2020 to 59.5% (n = 144) in 2024. Concurrently, the prevalence of females reporting one binge drinking episode per month increased substantially from 13.0% (n = 38) to 25.2% (n = 61). Smaller increases were also observed in higher-frequency binge drinking categories, including 3–5 times and >10 times per month, although these categories remained relatively uncommon overall.

Overall, these findings indicate a shift toward increased engagement in binge drinking behaviors among females between 2020 and 2024, whereas binge drinking patterns among males remained relatively stable over time, as presented in Table 5.

Although the reported drug consumption was low, illicit drug use declined markedly between 2020 and 2024 (*p* < 0.001), from 23.1% to 7.2% among males and from 21.2% to 5.0% among females. Table 6 presents illicit drug consumption among participants.

### 3.3. Physical Activity

Daily walking (≥30 min) increased substantially between 2020 and 2024 (*p* < 0.001), with 69.9% of males and 75.2% of females reporting daily activity in 2024, compared with no participants reporting daily walking in 2020. Similarly, engagement in moderate and vigorous physical activity (≥10 min/day) improved across both genders (*p* < 0.001), as presented in Table 7.

On the other hand, the results of the study evidenced that moderate physical activity (at least 10 min) showed a shift in frequency distribution between the pandemic and post-pandemic periods, with an increase in daily moderate physical activity reported in 2024. During the pandemic, 90.2% of the males and 84.7% of the females exercised moderately with a higher frequency (1–3 or 4–6 times per week) (Table 7).

Moreover, the majority of males and females were engaged 1–3 times per week in vigorous physical activity during the pandemic period. However, after the pandemic the frequency of vigorous physical activity changed across frequency categories, particularly among female students (Table 7).

### 3.4. Fast-Food Consumption

The results of the present study showed that fast-food consumption increased significantly across survey years (*p* < 0.001). The proportion of students reporting different frequencies of fast-food ingestion increased in both males and females during the post-pandemic period (Table 8).

### 3.5. Association Between Sleep and Other Factors

We run a multivariate analysis to predict factors that influence sleep quality among students.

The multiple linear regression model was statistically significant, F (4, 441) = 7.882, *p* < 0.05, with an R^2^ of 0.258. This indicates that 25.8% of the variance in sleep quality scores was explained by academic performance, drinking, gender, and drug use. While this represents a moderate effect size according to Cohen’s guidelines, the majority of the variance in sleep quality remains unexplained by the current model, suggesting that other variables may also play an important role (Table 9). In this model the quality of sleep was negatively associated with academic results, and associated with gender; females have a higher score, having a lower sleep quality (Table 9).

## 4. Discussion

This study assessed lifestyle behaviors, sleep quality, and health-related factors among university students in 2020, during the pandemic, and in 2024, post-pandemic, revealing significant changes over time in physical activity, sleep patterns, and substance use. Overall, the findings indicate that although some aspects of lifestyle, such as physical activity and reduced substance use, improved, sleep quality and body weight status worsened slightly during the same period.

The academic years of medical education are characterized by sustained academic demands, high workload, and continuous performance pressure, all of which may substantially affect students’ physical and psychological well-being. In particular, these stressors have been shown to disrupt sleep patterns, contributing to reduced sleep duration, impaired sleep quality, and irregular sleep–wake cycles. Decreased academic performance is associated with sleep disturbance [32]. Data from the literature regarding sleep quality among genders is controversial. On one hand, a higher prevalence of poor gender symptoms of anxiety, depression and stress was observed, especially among female students [33,34]. On the other hand, other studies found no significant differences between male and female medical students regarding sleep quality [35].

The results of the present study showed that female students experienced poor sleep quality and insufficient sleep duration, especially during the post-pandemic period. These findings suggest that attending an in-person university is more stressful than attending classes remotely.

The demanding and overwhelming load of work encountered by medical students and young doctors early in their careers not only affects the duration and quality of their sleep directly but also affects sleep by the buildup of stress indirectly. This creates a continuous vicious cycle of poor sleep, more stress, and less academic achievement.

### 4.1. Sleep Quality and Insomnia

The mean Athens Insomnia Scale scores increased significantly between 2020 and 2024, suggesting a higher prevalence of insomnia symptoms. Sleep deprivation among university students is well-documented and may be linked to increased academic stress, irregular schedules, and excessive screen time [36,37].

Interestingly, our findings indicate that sleep quality not only failed to recover in the post-pandemic period but further deteriorated, despite improvements in certain lifestyle factors such as physical activity. This paradoxical trajectory challenges the assumption that sleep disturbances observed during the pandemic were merely situational responses to lockdown measures. Instead, our results suggest a sustained, potentially cumulative impact of the pandemic on sleep health, particularly within a high-risk academic population such as medical students.

Sleep disturbances have also been widely reported among students from other academic disciplines, suggesting that poor sleep quality is a broader phenomenon within university populations. Studies conducted in general student samples during and after the COVID-19 pandemic have documented significant declines in sleep quality, often associated with increased stress, reduced physical activity, and changes in daily routines [38,39]. However, evidence indicates that medical students may experience a greater burden of sleep disturbances compared to their non-medical peers, likely due to the cumulative effects of intensive academic demands, competitive environments, and early clinical exposure [37,40,41]. While some studies report similar trends across disciplines, the magnitude and persistence of sleep impairment appear to be more pronounced among medical students [40,41]. These findings suggest that although sleep disruption is common among university students, medical training may act as an amplifying factor, contributing to more severe and sustained sleep-related difficulties.

### 4.2. Alcohol and Substance Use

The present study identifies changes in alcohol consumption patterns among medical students between the first wave of the COVID-19 pandemic (2020, confinement period) and the post-pandemic period (2024). Although overall drinking frequency decreased—particularly among female students—patterns of episodic consumption evolved in a more complex manner. While male students showed no significant change in weekly drinking frequency, female students exhibited a statistically significant shift toward lower frequency in 2024 compared to the confinement period of 2020. Specifically, abstinence rose, and weekly consumption declined among females. In our sample, female students demonstrated a significant increase in abstinence and a decrease in weekly alcohol consumption after the pandemic, whereas male students showed relatively stable drinking frequency. These findings partially align with previous studies conducted in university populations during lockdown periods. Studies from the literature reported a reduction in regular alcohol consumption among Spanish university students during confinement, largely attributed to reduced social gatherings and limited access to bars [42,43,44]. On the other hand, in Ireland, the restrictions produced mixed effects on the young population. Among these, 22.2% reported increased consumption, 17.2% reported decreased consumption, and 60.6% reported no change [45].

Despite reductions in weekly frequency and moderate-intensity drinking, our data revealed an increase in occasional binge drinking episodes among female students post-pandemic. Amaral et al. [46] found the same prevalence of excessive alcohol consumption among men and women, without significant differences in alcohol use between sexes. The factors contributing to our findings could include a shift in women’s societal roles and a trend toward gender equality, in which women invest more in education, work outside the home, adopt behaviors traditionally associated with men, and consume more alcohol, thereby reducing disparities in the consequences of alcohol use [46].

Gender differences in alcohol behavior among medical students are well documented. Evidence suggests that female students may be more vulnerable to stress-related or socially driven episodic drinking, particularly during transitional or high-pressure periods [47]. The return to in-person academic demands and social normalization may have contributed to compensatory binge episodes rather than regular drinking patterns.

A significant decline in illicit drug use was observed between 2020 and 2024 in both male and female medical students. International evidence or other Romanian studies [48] reported reduced substance use among young people during and after the COVID-19 pandemic [49,50,51,52]. These changes were commonly attributed to social restrictions and increased health risk awareness. In contrast, smoking prevalence did not change significantly, supporting evidence that tobacco use is more resistant to short-term contextual changes due to nicotine dependence and stress-related coping behaviors [53]. Studies conducted among university populations have demonstrated that substance use and smoking are associated with poorer sleep quality, insomnia symptoms, and reduced academic performance [54].

### 4.3. Nutritional Status and Lifestyle Factors

The analysis of body mass index showed a significant upward trend in overweight and obesity rates from 2020 to 2024. These findings are in line with global trends reporting increasing BMI among young adults during the post-pandemic period [6,7]. It is possible that factors such as reduced meal regularity, stress-related eating, and sedentary behavior could explain this shift.

Additionally, the findings of the study indicate a marked shift in fast-food consumption patterns among medical students from the pandemic period (2020) to the post-pandemic period (2024), characterized by a significant reduction in the proportion of students who reported never consuming fast food and a concomitant increase in moderate-to-high consumption frequencies. These changes were observed in both male and female students, suggesting a general post-pandemic normalization—or even intensification—of fast-food intake. Furthermore, the increase in fast-food consumption observed in the post-pandemic period was accompanied by significant modifications in both BMI distribution (*p* = 0.001) and physical activity patterns (*p* < 0.001). Specifically, between 2020 and 2024, the prevalence of overweight nearly doubled (10.9% vs. 19.1%), and the prevalence of obesity also increased (3.4% vs. 6.2%), while the proportion of normal-weight students declined (74.0% vs. 68.3%).

Furthermore, lifestyle restructuring following the pandemic—resumption of in-person academic activities, increased time pressure, and greater accessibility of food vendors—may have promoted convenience-oriented dietary behaviors.

On the other hand, post-pandemic data revealed an increase in reported physical activity levels. Although enhanced activity is generally associated with improved metabolic health, the concurrent rise in fast-food consumption may have offset these benefits. Frequent consumption of energy-dense fast food has been consistently linked to higher total energy intake, poorer diet quality, and greater adiposity in young adults, independent of physical activity levels [55]. In cross-sectional and longitudinal studies, fast-food intake has been associated with increased body weight, BMI, and cardiometabolic risk factors, likely due to high saturated fat and added sugar content and low micronutrient density [56].

### 4.4. Physical Activity

A substantial improvement was observed in physical activity levels. The COVID-19 home confinement had a negative effect on all PA intensity levels (vigorous, moderate, walking and overall). Additionally, daily sitting time increased from 5 to 8 h per day [57]. In the present study, the proportion of students reporting daily walking (≥30 min) or engaging in moderate and vigorous physical activity (≥10 min per day) increased significantly between 2020 and 2024. Studies from the literature showed that higher levels of physical activity improve sleep quality in university students [40,58]. Previous studies have shown that physical exercise relieves stress and negative emotions by secreting serotonin and dopamine, and promotes mental health [59]. The reduction of psychological stress and negative emotions helps improve sleep quality [60]. According to review studies, moderate physical activity benefited young people’s sleep quality. The stress-recovery theory of physical activity suggests that moderate activity disrupts homeostasis and the body initiates self-repair, which involves neuroendocrine regulation and is related to sleep regulation, supporting the effect of physical activity on sleep quality [61]. Additionally, female students have a greater probability of having insufficient levels of physical activity than male students [62,63].

On the other hand, physical activity helps get better academic performance by increased secretion of serotonin, improved cerebral circulation, hormone levels changes, and increased self-esteem [64,65]. Obese university students are more susceptible to psychological problems such as anxiety, depression and more likely to be socially isolated, which in turn influences their academic attainment. Furthermore, regular physical activity prevent weight gain in university students, a population especially at risk of sedentary behavior, as much of their campus day consists of classroom lectures and studying sitting still [66].

The observed increase in physical activity levels in 2024 compared to 2020 may reflect the transition from the COVID-19 confinement period to the post-pandemic context. In 2020, public health restrictions, limited access to recreational facilities, and reduced mobility likely constrained opportunities for regular exercise. By contrast, the lifting of restrictions in 2024 may have facilitated greater engagement in structured and habitual physical activity, contributing to the improvements observed across all intensity levels.

### 4.5. Association Between Sleep and Other Variables

The multiple regression analysis identified academic performance and gender as significant predictors of sleep quality. Poorer academic results and female gender were associated with higher insomnia scores, consistent with evidence showing that women report more sleep disturbances and are more vulnerable to stress-related sleep impairment [67,68]. Although alcohol and drug use were not significant predictors in the regression model, their long-term impact on sleep and cognitive function should not be underestimated.

Overall, the findings suggest that lifestyle behaviors among students are dynamic and influenced by multiple social, psychological, and environmental factors. While positive trends were observed in physical activity and substance use, deteriorations in sleep quality and BMI indicate that further strategies are required to promote balanced, sustainable health behaviors among university populations.

### 4.6. Limitations

The findings of this study should be interpreted in light of several limitations. First, the cross-sectional design does not allow causal inferences, but only the identification of associations between sleep quality and lifestyle-related factors [69]. Although data were collected at two distinct time points (2020 and 2024), participants were not followed longitudinally, which limits the interpretation of changes at the individual level.

Second, self-reported data may be subject to recall bias and social desirability bias, particularly for sensitive behaviors such as alcohol consumption, smoking, illicit drug use, and sleep duration [70]. Conducting questionnaires online addressed many of these limitations. In addition, sleep duration and physical activity were assessed subjectively, without objective measurements, which may have affected the precision of the estimates. Online completion of questionnaires limited these limitations.

The use of a convenience sample composed exclusively of medical students from a single geographical region may limit the generalizability of the results, while the unequal sex distribution could have influenced some sleep-related outcomes [71].

The lack of a comparison group from other academic disciplines limits our ability to directly assess whether the observed changes are specific to medical students or reflect a broader student population trend.

Furthermore, the two survey periods differed substantially in contextual conditions, with the 2020 assessment conducted during COVID-19 restrictions and the 2023–2024 survey reflecting a return to in-person education, which may have contributed to the observed differences [38,39].

Despite these limitations, the study has several important strengths, including a relatively large sample size, the use of a validated instrument for insomnia assessment (Athens Insomnia Scale), and the detailed evaluation of health-related behaviors. Moreover, the comparison of two distinct time points before and after the COVID-19 pandemic provides relevant insights into changes in sleep and lifestyle patterns among medical students.

## 5. Conclusions

The study highlights significant shifts in student lifestyle behaviors between 2020 and 2024. Improvements in physical activity and reductions in substance use contrast with worsening sleep quality and increased BMI. These findings underscore the need for targeted health promotion programs focusing on sleep hygiene, nutrition education, and stress management among university students.

### Future Research

Future research should further explore the long-term evolution of sleep patterns and associated lifestyle behaviors among university students, particularly in the post-pandemic context. Longitudinal studies would be valuable to assess whether the observed changes persist over time or represent temporary adaptations. In addition, qualitative approaches, such as interviews or focus groups, could provide deeper insight into students’ perceptions, coping strategies, and the contextual factors influencing sleep behaviors. Such methods may help to better understand the underlying mechanisms of sleep disturbances and inform the development of targeted, student-centered interventions.

Such research offers a valuable basis for designing comprehensive, multidimensional interventions targeting sleep hygiene, nutrition, physical activity, and alcohol consumption, with the potential to enhance health outcomes among Romanian medical students.

## Figures and Tables

**Table 1:** General characteristics of the sample.

Variable		2020 No, (%)	2024 No, (%)
Academic performance	Excellent	28 (7.3)	38 (11.70)
	Good	246 (64.1)	204 (62.80)
	Medium	105 (27.3)	79 (24.30)
	Bad	5 (1.3)	4 (1.20)
Academic year	1	95 (24.7)	21 (6.5)
	2	105 (27.3)	84 (25.8)
	3	27 (7.0)	89 (27.4)
	4	28 (7.3)	40 (12.3)
	5	90 (23.4)	57 (17.5)
	6	39 (10.2)	34 (10.5)
Place of living	Dormitorium	58 (15.1)	139 (42.8)
	Alone, in rent apartment	42 (10.9)	62 (19.1)
	Rent apartment with friends	65 (16.9)	42 (12.9)
	With the parents	168 (43.8)	38 (11.7)
	With a partner	51 (13.3)	44 (13.5)
Marital status	Single	197 (51.3)	245 (75.4)
	Married	6 (1.6)	0
	In a relationship	181 (47.1)	80 (24.6)
BMI kg/m^2^	Underweight	45 (11.7)	21 (6.5)
	Normo-weight	284 (74.0)	222 (68.3)
	Overweight	42 (10.9)	62 (19.1)
	Obese	13 (3.4)	20 (6.2)

**Table 2:** Sleep characteristics among students.

Year	2020	2024	2020	2024	
Gender	Male (Mean ± SD)	Male (Mean ± SD)	Female (Mean ± SD)	Female (Mean ± SD)	*p*-Value *
No. h sleep during session	7.27 ± 0.93	6.77 ± 1.55	7.29 ± 0.97	6.5 ± 1.35	0.08
No. h sleep outside the session	7.45 ± 1.35	7.12 ± 1.33	6.77 ± 1.23	7.61 ± 4.47	0.009
Athens score	4.05 ± 3.09	4.45 ± 3.16	4.5 ± 3.09	5.49 ± 3.18	0.01

* *t* test.

**Table 3:** Sleep quality among students.

Year	2020	2024	2020	2024	*p*-Value *
Gender	Male No, (%)	Male No, (%)	Female No, (%)	Female No, (%)	
Sufficient Sleep—No	14 (15.4)	13 (15.7)	57 (19.5)	39 (16.1)	0.383
Sufficient Sleep—Yes	77 (84.6)	70 (84.3)	236 (80.5)	203 (83.9)	
Rested in the morning—No	34 (37.4)	33 (39.8)	158 (53.9)	130 (53.7)	0.967
Rested in the morning—Yes	57 (62.6)	83 (60.2)	135 (46.1)	112 (46.3)	

* Chi Square.

**Table 4:** The prevalence of insomnia among students.

Parameter	Male No. (%)	Male No. (%)		Female No. (%)	Female No. (%)	
	2020	2024	*p*-Value	2020	2024	*p*-Value *
Athens score < 6	73 (80.2)	81 (73.5)	0.62	228 (77.8)	168 (69.4)	0.48
Athens score ≥ 6	18 (19.8)	22 (26.5)		65 (22.2)	74 (30.6)	

* Chi Square.

**Table 5:** Alcohol consumption among students.

Parameter	2020	2024		2020	2024	
Frequency of Alcohol Consumption	Male (No (%))		*p*-Value *	Female (No (%))		*p*-Value *
Not at all	22 (24.2)	27 (32.5)	0.31	98 (33.4)	116 (47.9)	0.002
1–4 times/week	68 (74.7)	55 (66.3)		189 (64.5)	122 (50.4)	
5–6 times/week	0	1 (1.2)		3 (1.0)	4 (1.7)	
Every day	1 (1.1)	0		3 (1.0)	0	
No. of drinks per row						
Not at all	8 (8.8)	20 (24.1)	0.02	52 (17.7)	65 (26.9)	0.01
1–3 drinks	45 (49.5)	39 (47.0)		185 (63.1)	149 (61.6)	
4–7 drinks	32 (35.2)	18 (21.7)		53 (18.1)	27 (11.2)	
>8 drinks	6 (6.6)	6 (7.2)		3 (1.0)	1 (0.4)	
Excessive alcohol consumption frequency						
None	49 (53.8)	40 (48.2)	0.62	220 (75.1)	144 (59.5)	0.001
Once	19 (20.9)	15 (18.1)		38 (13)	61 (25.2)	
2 times	12 (13.2)	13 (15.7)		25 (8.5)	19 (7.9)	
3–5 times	8 (8.8)	7 (8.4)		8 (2.7)	14 (5.8)	
6–9 times	2 (2.2)	6 (7.2)		1 (0.3)	1 (0.4)	
>10 times	1 (1.1)	2 (2.4)		1 (0.3)	3 (1.2)	

* Chi Square.

**Table 6:** Illicit drug consumption and smoking among students.

Parameter	Male No. (%)		*p*-Value *	Female No. (%)		*p*-Value *
	2020	2024		2020	2024	
Illicit drug consumption						
No	70 (76.9)	77 (92.8)	0.004	231 (78.8)	230 (95.0)	<0.001
Yes	21 (23.1)	6 (7.2)		62 (21.2)	12 (5.0)	
Smoking						
No	64 (70.3)	65 (78.3)	0.23	217 (74.1)	187 (77.3)	0.39
Yes	27 (29.7)	27 (21.7)		76 (25.9)	55 (22.7)	

* Chi Square.

**Table 7:** Physical activity of the sample.

Parameter	Male No. (%)			Female No. (%)		
	2020	2024	*p*-Value *	2020	2024	*p*-Value *
**Walking**						
Never	9 (9.9)	0	0 < 0.001	52 (17.7)	0	<0.001
1–3 times/week	43 (47.3)	5 (6.0)		173 (59.0)	8 (3.3)	
4–6 times/week	39 (42.9)	20 (24.1)		68 (23.2)	52 (21.5)	
Every day	0	58 (69.9)		0	185 (75.2)	
Moderate PA						
Never	9 (9.9)	7 (8.4)	<0.001	45 (15.4)	36 (14.9)	<0.001
1–3 times/week	43 (47.3)	32 (38.6)		176 (60.1)	129 (15.3)	
4–6 times/week	39 (42.9)	19 (22.9)		72 (24.6)	43 (17.8)	
Every day	0	25 (30.1)		0	34 (14.0)	
Vigorous PA						
Never	24 (26.4)	10 (12)	0.002	83 (28.3)	61 (25.2)	<0.001
1–3 times/week	45 (49.5)	33 (39.8)		164 (56.0)	135 (55.8)	
4–6 times/week	22 (24.2)	22 (26.5)		46 (15.7)	30 (12.4)	
Every day	0	18 (21.7)		0	16 (6.6)	

* Chi Square.

**Table 8:** Fast food consumption of the sample.

Parameter	Male No. (%)			Female No. (%)		
Fast Food Frequency	2020	2024	*p*-Value *	2020	2024	*p*-Value *
never	25 (27.5)	6 (7.2)	0.002	74 (25.3)	18 (7.4)	<0.001
sometimes	48 (52.7)	49 (59.0)		179 (61.1)	178 (73.6)	
1–3 times/week	16 (17.6)	20 (24.1)		36 (12.3)	42 (17.4)	
4–6 times/week	92 (2.2)	8 (9.6)		217 (74.1)	187 (77.3)	

* Chi Square.

**Table 9:** Sleep quality scores and academic performance regression.

Variables	B	Std. Error	Beta	T	*p*	Tolerance	VIF
(Constant)	6.359	0.327		19.436	000	0.971	1.03
Academic results (excellent, good vs. medium, bad)	−1.569	0.338	−0.216	−4.636	<0.001	0.994	1.006
Alcohol consumption (Yes/No)	0.208	1.577	0.006	0.132	0.895	0.979	1.021
Illicit drugs	−0.551	0.373	−0.069	−1.476	0.141	0.992	1.008
Gender (male vs. female)	−1.133	0.331	−0.158	−3.416	0.001	0.971	1.03

Dependent Variable: sleep quality score; *p* < 0.05 was considered statistically significant.

## Data Availability

The datasets generated and analyzed during the current study are not publicly available, since they were specifically collected by the authors for the present study, but may be available from the corresponding author on reasonable request.

## Supplementary Materials

The following supporting information can be downloaded at: https://www.mdpi.com/article/10.3390/medicina62050880/s1. Table S1. The prevalence of insomnia among students.
