# Supplementary material for: Sleep Quality and Associated Lifestyle Factors Among Medical Students Before and After the COVID-19 Era—A Comparative Study from Romania
Source: Medicina (Kaunas). 2026 May 4;62(5):880. doi: 10.3390/medicina62050880 (PMC13209030; doi:10.3390/medicina62050880)
Supplement: Supplementary file 1 [file medicina-62-00880-s001.zip › medicina-4217695-supplementary.pdf]

Item-level AIS responses by gender and survey year on Athens Insomnia Scale

Table S1. The prevalence of insomnia among students.

Year	2020	2024		2020	2024	
**Gender**	**Male No. (%)**		***p value***	**Female No. (%)**		***p value****
Sleep induction						
No problem	41 (45.1)	32 (38.6)	0.27	104 (35.5)	91 (37.6)	0.13
Slightly delayed	39 (42.9)	37 (44.6)		133 (45.4)	120 (49.6)	
Markedly delayed	9 (9.9)	14 (16.9)		42 (14.3)	27 (11.2)	
Very delayed /not sleep	2 (2.2)	0 (-)		14 (4.8)	4 (1.7)	
Awakenings during the night						
No problem	76 (83.5)	58 (69.9)	0.05	204 (69.6)	132 (54.5)	0.002
Minor Problem	13 (14.3)	18 (21.7)		65 (22.2)	132 (34.3)	
Considerable problem	2 (2.2)	7 (8.4)		21 (7.2)	26 (10.7)	
Serious problem/not sleep at all	0	0		3 (1.0)	1 (0.4)	
Final awakening earlier than desired						
Not earlier	75 (82.4)	58 (69.9)	0.05	242 (82.6)	171 (70.7)	0.006
A little earlier	12 (13.2)	23 (27.7)		43 (14.7)	65 (26.9)	
Markedly earlier	4 (4.4)	2 (2.4)		6 (2.0)	4 (1.7)	
Much earlier, or did not sleep at all	0	0		2 (0.7)	2 (0.8)	
Total sleep duration						
Sufficient	54 (59.3)	34 (41.0)	0.06	154 (52.6)	97 (40.1)	0.012
Slightly insufficient	28 (30.8)	39 (47.0)		125 (42.7)	124 (51.2)	
Markedly insufficient	8 (8.8)	10 (12.0)		14 (4.8)	19 (7.9)	

Very insufficient or did not sleep at all	1 (1.1)	0		0	2 (0.8)	
Overall quality of sleep						
Satisfactory	57 (62.6)	50 (60.2)		172 (58.7)	105 (43.4)	
Slightly unsatisfactory	26 (28.6)	28 (33.7)	0.65	106 (36.2)	116 (47.9)	0.004
Markedly unsatisfactory	8 (8.8)	5 (6.0)		12 (4.1)	18 (7.4)	
Very unsatisfactory or did not sleep at all	0	0		3 (1.0)	3 (1.2)	
Sense of well-being during the day						
Normal	60 (65.9)	68.7 (57)		198 (67.6)	128 (52.9)	
Slightly decreased	23 (25.3)	22 (26.5)	0.57	88 (30.0)	93 (38.4)	<0.001
Markedly decreased	8 (8.8)	4 (4.8)		7 (2.4)	17 (7.0)	
Very decreased	0	0		0	4 (1.7)	
Functioning (physical and mental) during the day						
Normal	55 (60.4)	49 (59.0)	0.01	169 (57.7)	109 (45.0)	<0.001
Slightly decreased	28 (30.8)	28 (33.7)		107 (36.5)	117 (48.3)	
Markedly decreased	0	5 (6.0)		0	14 (5.8)	
Sleepiness during the day						
Very decreased	8 (8.8)	1 (1.2)		17 (5.8)	2 (0.8)	
None	20 (22.0)	18 (21.7)		23 (7.8)	22 (9.1)	
Mild	54 (59.3)	53 (63.9)	0.87	214 (73.0)	144 (59.5)	0.004
Considerable	16 (17.6)	11 (13.3)		51 (17.4)	64 (26.4)	
Intense	1 (1.1)	1 (1.2)		5 (1.7)	12 (5.0)	

*Chi Square
